# Modified Global and Modified Linear Contrast Stretching Algorithms: New Colour Contrast Enhancement Techniques for Microscopic Analysis of Malaria Slide Images

**DOI:** 10.1155/2012/637360

**Published:** 2012-10-03

**Authors:** Aimi Salihah Abdul-Nasir, Mohd Yusoff Mashor, Zeehaida Mohamed

**Affiliations:** ^1^Electronic & Biomedical Intelligent Systems (EBItS) Research Group, School of Mechatronic Engineering, Universiti Malaysia Perlis, Campus Pauh Putra, Perlis, 02600 Pauh, Malaysia; ^2^Department of Medical Microbiology & Parasitology, School of Medical Sciences, Health Campus, Universiti Sains Malaysia, 16150 Kubang Kerian, Kelantan, Malaysia

## Abstract

Malaria is one of the serious global health problem, causing widespread sufferings and deaths in various parts of the world. With the large number of cases diagnosed over the year, early detection and accurate diagnosis which facilitates prompt treatment is an essential requirement to control malaria. For centuries now, manual microscopic examination of blood slide remains the gold standard for malaria diagnosis. However, low contrast of the malaria and variable smears quality are some factors that may influence the accuracy of interpretation by microbiologists. In order to reduce this problem, this paper aims to investigate the performance of the proposed contrast enhancement techniques namely, modified global and modified linear contrast stretching as well as the conventional global and linear contrast stretching that have been applied on malaria images of *P. vivax* species. The results show that the proposed modified global and modified linear contrast stretching techniques have successfully increased the contrast of the parasites and the infected red blood cells compared to the conventional global and linear contrast stretching. Hence, the resultant images would become useful to microbiologists for identification of various stages and species of malaria.

## 1. Introduction

 Malaria is a widely prevalent disease, affecting millions of people in the world. Research has shown that malaria is caused by a protozoan parasite of the genus *Plasmodium*. *Plasmodium* is a small, single-cell organism which originated from a species of mosquito named *Anopheles* [1]. Malaria is passed on by the female *Anopheles* mosquito biting a person who has malaria parasites in their blood. If the disease is not treated, it can lead to serious problems such as anemia, retinal damage, and convulsions [2]. Malaria infection ends in one of two ways either the patient dies or the parasites are defeated by immune system or medications. Up to this date, five species of genus *Plasmodium* that can cause human infection have been discovered namely, *P. falciparum*, *P. vivax*, *P. ovale*, *P. malariae,* and *P. knowlesi *[3]. In 2009, it is estimated that 3.3 billion people which is half of the world population are at risk of malaria, with approximately 225 million cases and 781,000 of deaths [4]. The vast majority of cases occur in children under the age of 5 and also pregnant women. In Malaysia, a total of 7,010 malaria cases were diagnosed among Malaysians in 2009 [4].

 Diagnosis of malaria must be prompt, as a few hours of delay in treatment involves a matter of life and death. Currently, there are several new methods have been employed for diagnosis of malaria. These include the use of fluorescent microscopy, rapid antigen detection method, and polymerase chain reaction (PCR) technique [5]. Despite these advances, the most economic and reliable diagnosis which is based on microscopic examination of blood slide remains the gold standard for laboratory confirmation of malaria [5, 6].The procedure is performed manually by expert microbiologists through visual identification under light microscope [6]. The percentage of parasitaemia as well as the species and life-cycle stages of the detected parasite must be determined in order to provide the best treatment. The scheme that requires the examination of both thick and thin blood smears for the presence of plasmodia is believed to be the most sensitive and specific one [7]. Besides these numerous advantages, there are also some limitations associated with the identification of malaria based on light microscopy, such as time consumption and labour intensity. In addition, the accuracy of the final diagnosis varies depending on the skill and experience of the experts [7, 8]. In comparison to the expert microscopy, standard laboratory microscopy has a sensitivity of approximately 90%, a figure which drops dramatically in the field [7]. In Malaysia, there are more than 1.5 million slides examined each year in a population at risk of approximately 1 million [3]. Due to human error and time consumption, better and more efficient method is needed.

 The contrast of the malaria image is one of the factors that may influence the accuracy of interpretation by microbiologists. The malaria slide images that have been captured through the microscope may have their own weaknesses such as blurred or low contrast due to the magnification or underexposure of the light built in the system of the image analyzer. In addition, variable smears quality and the conditions of the slide are highly influenced by the time and storage. Due to the low quality of the image, it will be hard to visualize and analyze the morphological features between the different plasmodia species on the screen, hence increasing the false diagnosis rate. Thus, contrast enhancement technique at the preprocessing stage is developed to adjust the quality of image for better human visual perception [9]. The resulting enhanced medical image will provide clearer and cleaner images for better and easier disease screening process by the doctor.

 Various approaches of contrast enhancement techniques have been developed for enhancing the image contrast. Despite of histogram equalization, contrast stretching is one of the popular contrast enhancement technique that has been applied in X-ray [10] and various medical images such as leukaemia [11], retinal fundus [12], and computed tomography (CT) brain images [13]. There are several contrast enhancement techniques that have been developed for malaria image. Tek [14] proposed a new colour normalization method that has been applied on peripheral thin-film blood images. This method has been developed in order to maintain the colour constancy of the images that have been captured from various sources. The normalization has been applied separately to the foreground and background regions. A rough estimation of the foreground-background regions is done by mathematical morphology and followed by a refined segmentation using histograms of these regions. Then, an illumination-independent response is calculated using the background region. The normalization is completed by transforming the foreground region according to the grey values determined by a reference set. The proposed method has been tested on various images and has been found successful.

 Another example for the application of contrast enhancement technique is the use of dark stretching technique for enhancing and segmenting the *P. falciparum* based on thick blood smear images [15]. By applying the dark stretching technique, the darkest region of the image which is referred to the parasite will be stretched, while the bright region will be compressed. Thus, the appearance of the parasites will become clearer due to the stretching process in dark stretching technique. Due to the requirement for enhancing the malaria image, the current study investigates the performance of contrast enhancement techniques namely, global and linear contrast stretching as well as the proposed modified global and modified linear contrast stretching that have been applied to enhance the malaria slide images. The proposed contrast enhancement technique is expected to improve the performance of microscopy by improving the quality and clarity especially on degrading or low contrast malaria image as well as easing the image segmentation process in the later part of the diagnostic system.

## 2. Morphological Features of Malaria

**Figure 1:**
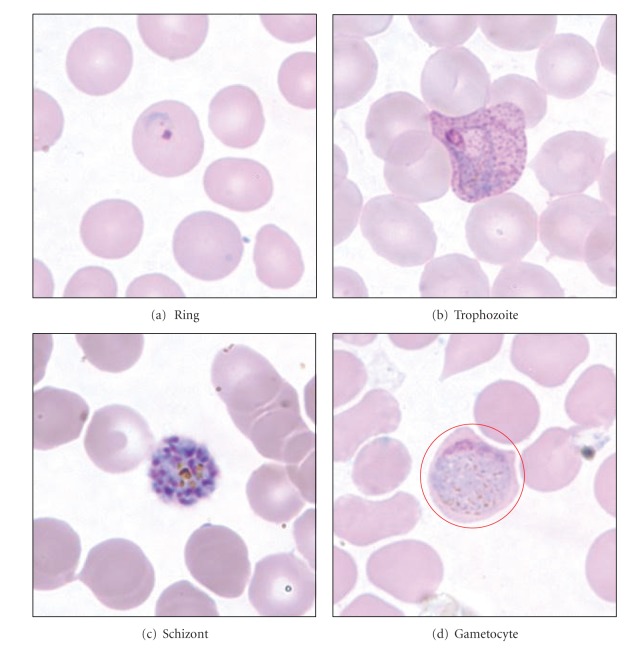
The malaria images for the four life-cycle stages of *P. vivax* [16].

Generally in malaria diagnosis, the process is performed by searching for the parasites in blood slide through a microscope. The visual aspect of the parasites and the red blood cells (RBCs) can be distinguished based on colour due to the used of chemical named Giemsa stain [1]. Then, specific morphological features will be observed in order to identify the stages and species of malaria. During the life-cycle in peripheral blood, the five malaria species may be observable in the four different life-cycle stages which are morphologically distinguishable between the ring, trophozoite, schizont, and gametocyte [1]. In this study, the four contrast enhancement techniques have been applied on four life-cycle stages of *P. vivax* images. The malaria images for the four life-cycle stages of *P. vivax* are shown in Figure 1.

Here, The variations of malaria parasite morphology that are generally being observed are as follows [1, 16];

- size: the size of the parasite and the infected RBC (red circle);
- form: the configuration of the nucleus and cytoplasm of the parasite which occur during the growth of the parasite;
- colour: the fraction of the stain taken up by the various elements of the parasite;
- pigment: the presence or absence of malarial pigment.

## 3. Methodology

In this study, the proposed work comprises of three main steps. These include image acquisition, image enhancement, and measurement of image quality for the four contrast enhancement techniques

### 3.1. Image Acquisition

Image acquisition is the first stage of vision system and image processing area. In this study, 150 malaria images which consist of the ring, trophozoite, schizont, and gametocyte stages have been captured from eight different slides of thin blood smear from *P. vivax* samples. The malaria slides are prepared by the Medical Microbiology & Parasitology Department, Hospital University Science Malaysia (HUSM). The slides are examined under 100x oil immersion objective using Leica DLMA microscope. The images are then captured using Infinity-2 camera at a resolution setting of 800 × 600 pixels and saved in bitmap (*.bmp) format. The captured images are studied under the supervision of microbiologists in order to recognize and differentiate between the four life-cycle stages of *P. vivax* species.

### 3.2. Contrast Enhancement Techniques

The malaria slide images captured through the microscope may have their own weaknesses such as blurred or low contrast. Thus, contrast enhancement technique plays an important role in enhancing the quality and contrast of malaria images. In general, an image can be enhanced by spreading the range of colour values to make use of all possible values. This method is called contrast stretching. It changes the distribution and range of the digital numbers assigned to each pixel in an image. Here, there are four types of contrast stretching methods that have been applied on malaria images namely global, linear, modified global, and modified linear contrast stretching techniques.

#### 3.2.1. Global and Linear Contrast Stretching

 Global contrast stretching (GCS) technique remedies problems that manifest themselves in a global fashion such as excessive or poor lightning conditions in the source environment [17]. This technique enhances the image from the luminance information of an entire image. Image with a high global contrast will cause a global feeling of a detailed and variation-rich image. On the other hand, image with a lower global contrast contain less information, less details and appears to be more uniform [18].

The formula for global contrast stretching is governed by the following equation [19]:

(1) $$\begin{array}{c} {\text{out}}_{\text{RGB}}\left(x,y\right)=255*\left[\frac{\left({\text{in}}_{\text{RGB}}\left(x,y\right)-\min_{\text{RGB}}\right)}{\max_{\text{RGB}}-\min_{\text{RGB}}}\right], \end{array}$$

where in_RGB_(*x*, *y*) is The original RGB value of the pixel, out_RGB_(*x*, *y*) is the new RGB value of the pixel, min⁡_RGB_ is minimum value between the RGB components, and max⁡_RGB_ is maximum value between the RGB components

Based on (1), (*x*, *y*) is the image pixel location. min⁡_RGB_ and max⁡_RGB_ are the minimum and maximum values between the RGBs (red, green, and blue) of the original image. The GCS technique will consider all ranges of RGB colours at once to determine the minimum and maximum values between the RGB components. The combination between the RGB components will give only one value for each minimum and maximum parameter which will later be used for the contrast stretching process. However, the process for selecting these minimum and maximum values is different for the linear contrast stretching (LCS) technique.

 For LCS technique, the stretching process for each of the RGB components is carried out separately. Here, the amount of stretching that will be applied in a neighborhood will be controlled by the original contrast in that neighborhood. The formula for linear contrast stretching is governed by (2) [20]:

(2) $$\begin{array}{c} {\text{out}}_{\text{RGB}}\left(x,y\right)=255*\left[\frac{\left(\text{i}{\text{n}}_{\text{RGB}}\left(x,y\right)-\min\right)}{\max-\min}\right], \end{array}$$

where in_RGB_(*x*, *y*) is the original RGB value of the pixel, out_RGB_(*x*, *y*) is the new RGB value of the pixel, min⁡ is minimum value for each RGB components, and max⁡ is maximum value for each RGB component

Based on (2), the LCS technique will consider each range of RGB components in the image. Thus, the range of each colour component will be used during the contrast stretching process to represent each range of colour. This will give each colour component a set of minimum and maximum values. By applying this technique, each RGB component will be distributed linearly over the whole histogram so that the dynamic range of the histogram (0–255) is fulfilled [9].

#### 3.2.2. Modified Global and Modified Linear Contrast Stretching

 The enhancement of malaria images depends directly on the minimum and maximum values that will be used during the contrast stretching process. Here, new contrast enhancement techniques namely, modified global contrast stretching (MGCS) and modified linear contrast stretching (MLCS) have been proposed. Both techniques include a step to determine the new minimum and maximum values, which are beyond the original minimum and maximum values for each of the RGB components in the image. The modified global and modified linear contrast stretching techniques are similar to the minimum-maximum global and linear contrast stretching, except that these techniques use specified minimum and maximum values that lie in a certain percentage of pixels from the total number of pixels in the image.

In order to obtain the new minimum and maximum values for each of the RGB components in the image for both MGCS and MLCS techniques, several parameters are required during the calculation process. These include the value for minimum percentage, min⁡_*p*_, maximum percentage, max⁡_*p*_, number of pixels in each pixel level, *T*pix, total number of pixels that lie in a specified minimum percentage, *T*min⁡, and total number of pixels that lie in a specified maximum percentage, *T*max⁡. The procedures to develop the proposed MGCS and MLCS techniques are as follows.

(1) Select the desired values for min⁡_*p*_ and max⁡_*p*_.
(2) Initialize *T*min⁡  = 0 and *T*max⁡  = 0. Set the value of *k* = 0, where *k* is the current pixel level.
(3) Calculate the histogram for the red component.
(4) Obtain the number of pixels, *T*pix[*k*] at *k*. If *T*pix[*k*]≥1, set min⁡ = *T*min⁡+*T*pix[*k*].
(5) Check the following condition:
  (3) $$\begin{array}{c} \frac{T\min}{\text{Total}\;\text{number}\;\text{of}\;\text{pixels}\;\text{in}\;\text{the}\;\text{image}}*100\geq \min_{p}. \end{array}$$
(6) If *T*min⁡ satisfies (3), set the new minimum value, *N*min⁡ for the red component in the image to the *k* value that satisfies this condition; else set *k* = *k* + 1.
(7) Repeat steps 4 to 6 for the next pixel levels until *N*min⁡ is obtained based on the *k* value that satisfies (3).
(8) Set the value of *k* = 255.
(9) Obtain *T*pix[*k*] at *k*. If *T*pix[*k*]≥1, set *T*max⁡ = *T*max⁡ + *T*pix[*k*].
(10) Check the following condition:
  (4) $$\begin{array}{c} \frac{T\max}{\text{Total}\;\text{number}\;\text{of}\;\text{pixels}\;\text{in}\;\text{the}\;\text{image}}*100\geq \max_{p}. \end{array}$$
(11) If *T*max⁡ satisfies (4), set the new maximum value, *N*max⁡ for the red component in the image to the *k* value that satisfies this condition; else set *k* = *k* − 1.
(12) Repeat steps 9 to 11 for the next pixel levels until *N*max⁡ is obtained based on the *k* value that satisfies (4).
(13) Repeat steps 2 to 12 in order to calculate the *N*min⁡ and *N*max⁡ for the green and blue components.
(14) Determine the new minimum value between the RGB components, *N*min⁡_RGB_ and the new maximum value between the RGB components, *N*max⁡_RGB_ based on the *N*min⁡ and *N*max⁡ that have been obtained for each of the RGB components.
(15) For MGCS algorithm, substitute min⁡_RGB_ and max⁡_RGB_ in (1) with the *N*min⁡_RGB_ and *N*max⁡_RGB_ that have been obtained based on step 14.
(16) For MLCS algorithm, substitute min⁡ and max⁡ in (2) with the *N*min⁡ and *N*max⁡ that have been obtained for each of the RGB components.
(17) End.

**Figure 2:**
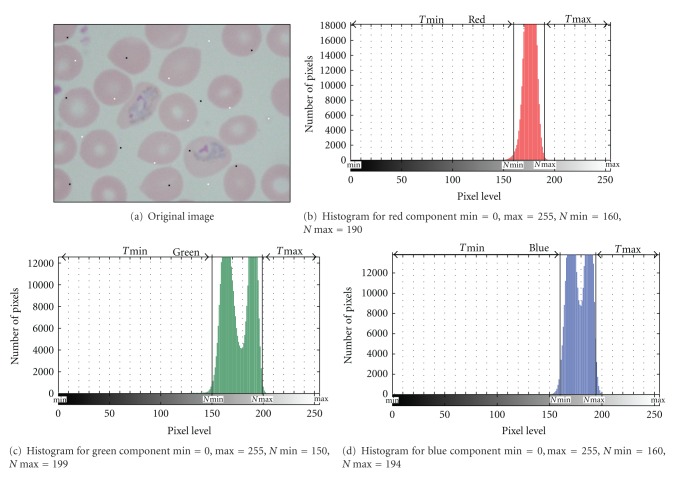
An example for determining the minimum and maximum values for each of the RGB components based on the proposed MGCS and MLCS techniques.

Figure 2 shows an example for determining the minimum and maximum values for each of the RGB components based on the proposed MGCS and MLCS techniques. Here, Figure 2(a) shows the original image of trophozoite stage with the addition of salt-and-pepper noise. Meanwhile, the histograms for the red, green, and blue components of the original image are shown in Figures 2(b), 2(c) and 2(d), respectively. By referring to Figure 2, it is found that all the minimum and maximum values for each of the RGB components are 0 and 255, respectively. Thus, these values are not suitable to be applied for the contrast stretching process. By applying the MGCS and MLCS techniques with min⁡_*p*_ and max⁡_*p*_ of 1%, the minimum and maximum values for each of the RGB components have been changed, and the *N*min⁡ and *N*max⁡ values can be referred to in Figure 2.

### 3.3. Quantitative Measure of Contrast Enhancement Techniques

**Figure 3:**
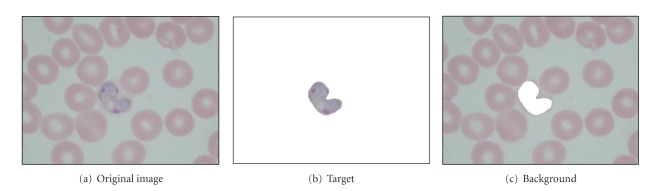
Original malaria image and its segmented parasite and background regions after performing the manual segmentation.

 Image quality measure has become crucial for most image processing applications. In general, the measurement of image quality can be divided into two primary ways which are qualitative and quantitative measures. In malaria image, the level of contrast improvement provided by the proposed contrast enhancement techniques is judged by the fact that it should increase the contrast between the target and background regions so that the target is visible against the background as well as keeping the colour structure of the original image. Here, the target is referred to the parasite.  Figure 3 shows the original malaria image and its segmented parasite and background regions after performing the manual segmentation.

 Generally, it is hard to state the level of enhancement by only depending on human visual interpretation. Thus, a quantitative measure of contrast enhancement is required in order to quantify the degree of contrast between these two regions. Based on this argument, a quantitative measure namely, distribution separation measure (DSM) which is based on the probability density function (PDF) of the target and the background regions, before and after enhancement, has been used for the quantitative analysis [21, 22]. By using DSM, a measure of separation between these two PDFs would be an indicator of the performance of the proposed contrast enhancement techniques on malaria images. The procedures to apply the quantitative measure of contrast enhancement techniques on malaria images are as follows.

(1) Apply the four contrast enhancement techniques which are GCS, LCS, MGCS, and MLCS on malaria images.
(2) Apply manual segmentation on both original and enhanced malaria images in order to obtain the target, *T* (parasite), and background, *B*, regions as shown in Figure 3.
(3) Calculate the best decision boundary for the original image between the target and background regions based on the following equation [21]:
  (5) $$\begin{array}{c} D1=\frac{\left(\mu_{B}^{O}\sigma_{T}^{O}\right)+\left(\mu_{T}^{O}\sigma_{B}^{O}\right)}{\left(\sigma_{T}^{O}+\sigma_{B}^{O}\right)}, \end{array}$$
 where *μ*_*T*_^*O*^, *σ*_*T*_^*O*^, *μ*_*B*_^*O*^, and *σ*_*B*_^*O*^ are the mean and standard deviation for each of the RGB components comprising the parasite and background regions for the original image, respectively.
(4) Calculate the best decision boundary for the enhanced image between the target and background regions based on the following equation [21]:
  (6) $$\begin{array}{c} D2=\frac{\left(\mu_{B}^{E}\sigma_{T}^{E}\right)+\left(\mu_{T}^{E}\sigma_{B}^{E}\right)}{\left(\sigma_{T}^{E}+\sigma_{B}^{E}\right)}, \end{array}$$
 where *μ*_*T*_^*E*^, *σ*_*T*_^*E*^, *μ*_*B*_^*E*^, and *σ*_*B*_^*E*^ are the mean and standard deviation for each of the RGB components comprising the parasite and background regions for the enhanced image, respectively.
(5) Calculate the value of DSM based on the following equation [21]:
  (7) DSM=(|D2−μBE|+|D2−μTE|) −(|D1−μBO|+|D1−μTO|).

A good enhancement technique must provide a DSM value greater than 0. The higher the DSM, the better separation between the distributions. Hence, the better is the enhancement technique. If the value of DSM is less than 0, there is no enhancement in the image [21].

## 4. Results and Discussions

In this study, the four contrast enhancement techniques namely, global, linear, modified global, and modified linear contrast stretching have been applied on 150 malaria images which consist of the ring, trophozoite, schizont, and gametocyte stages of *P. vivax* species. In order to access the significance of the enhancement technique on malaria image, the comparison between the original image and enhanced image is needed. For each enhancement technique, the qualities of images are initially evaluated based on human visual interpretation and then further analyzed by using a quantitative measure namely, distribution separation measure. 

### 4.1. Qualitative Analysis

 Exposure of a microscope as well as variable smears quality and the conditions of the slides are some factors that may influence the quality of the captured images. In order to assess the proposed work, the captured images with normal, blurred, and underexposure conditions have been processed using the proposed procedure. 

**Figure 4:**
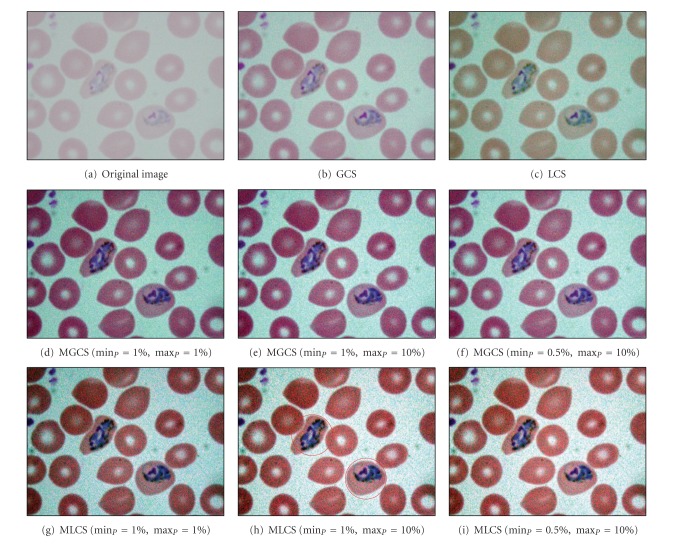
Results of contrast enhancement techniques for the blurred trophozoite image.

**Figure 5:**
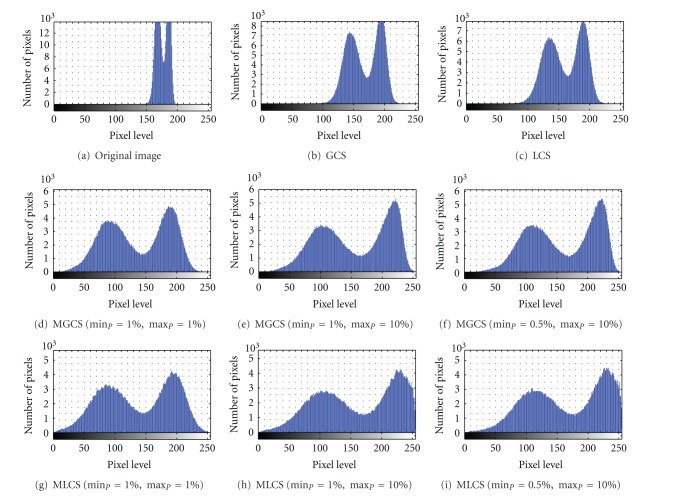
Intensity histograms for Blurred Trophozoite image after applying contrast enhancement techniques.

Figure 4(a) shows the original blurred image of trophozoite stage named as blurred trophozoite image. Based on this malaria image, the morphologies of the parasites are hardly be seen due to the blurred and low image contrast. The results of applying the four contrast enhancement techniques on blurred trophozoite image are shown in Figures 4(b)–4(i), while their corresponding intensity histograms are shown in Figures 5(b)–5(i).

 The result of applying the GCS technique is shown in Figure 4(b). Generally, this technique has produced an image which does not much differ in terms of changes in RGB colour from the original image. Based on the resultant image, there is a slight increase of contrast in malaria image. The GCS technique has very limited dynamic adjustment range. Thus, the resultant image has become slightly brighter compared to the original image. The result of applying the LCS technique on malaria image is shown in Figure 4(c). Based on the resultant image, there is also a slight increase of contrast in malaria image. The application of LCS technique will result in obtaining different colour structures between the RGB components inside the image. Based on the colour differences, the appearance of the parasite can easily be distinguished from the RBC and background regions.

 The results obtained after applying the proposed MGCS and MLCS techniques on malaria images are shown in Figures 4(d)–4(f) and Figures 4(g)–4(i), respectively. Based on the resultant malaria images, the images have become clearer and the contrast of the parasites, infected RBC and background regions has been successfully enhanced. In addition, the enlargement in terms of size for the infected RBC (red circle) which is one of the main malaria characteristics can easily be seen. Here, there are three different sets of minimum and maximum percentage values that have been used to determine the new minimum and maximum RGB values for contrast stretching process. The first set is min⁡_*P*_ = 1% and max⁡_*P*_ = 1%, the second set is min⁡_*P*_ = 1% and max⁡_*P*_ = 10%, and the third set is min⁡_*P*_ = 0.5% and max⁡_*P*_ = 10%. Here, the first, second, and third sets are named as Set I, Set II, and Set III, respectively. For the Set I, a small number of percentage value which is 1 has been used for both min⁡_*P*_ and max⁡_*P*_ in order to access the significance of the proposed MGCS and MLCS techniques on malaria images, compared to the conventional GCS and LCS techniques. Meanwhile, Set II and Set III have used different percentage values for both min⁡_*P*_ and max⁡_*P*_ in order to measure the effect of stretching process for the data that lie on the left and right sides of the histogram. By using these different percentage values for both MGCS and MLCS techniques, changes of contrast inside the resultant malaria images can be easily seen. Figures 4(d) and 4(g) show the resultant images after applying the MGCS and MLCS techniques with the min⁡_*P*_ = 1% and max⁡_*P*_ = 1%. By applying these values, the narrow range of data in original image will be stretched linearly to a wider range of data so that the dynamic range of the histogram (0 to 255) is fulfilled as shown in Figures 5(d) and 5(g). As a result, the contrast of malaria images has been enhanced compared to the contrast of the original image. Figures 4(e) and 4(h) show the resultant images after applying the MGCS and MLCS techniques with the min⁡_*P*_ = 1% and max⁡_*P*_ = 10%. Based on the resultant images, the contrast of malaria images has been enhanced and the background region has become brighter compared to the contrast of the original image. By applying the max⁡_*P*_ = 10%, the RGB values of the RBC and background regions have been shifted to the right side of the histogram. Thus, the narrow ranges of data in original image have been stretched to a wider range of data at the right side of the histogram. Based on the intensity histograms in Figures 5(e) and 5(h), pixels clustered at the right side of the histogram indicate that the resultant images become brighter. 

 Figures 4(f) and 4(i) show the resultant images after applying the MGCS and MLCS techniques with min⁡_*P*_ = 0.5% and max⁡_*P*_ = 10%. Based on the resultant images, both techniques have produced colour image with good image contrast compared to the contrast of the original image and the resultant images shown in Figures 4(d) and 4(g). By applying the min⁡_*P*_ = 0.5%, the RGB values of the parasite pixels that lie at the left side of the histogram have been shifted to the right side of the histogram. As a result, the parasite region has become brighter and the presence of malarial pigment inside the parasite can be easily seen. By comparing the three different sets of percentage values, the MGCS and MLCS techniques using Set II and Set III have produced the most acceptable results in enhancing the contrast of malaria images compared to the resultant images provided by Set I. Even though the dynamic range of the histogram has been fulfilled after applying the MGCS and MLCS techniques using Set I, the appearance of the parasite and RBC regions are darker compared to the resultant images provided by Set II and Set III.

**Figure 6:**
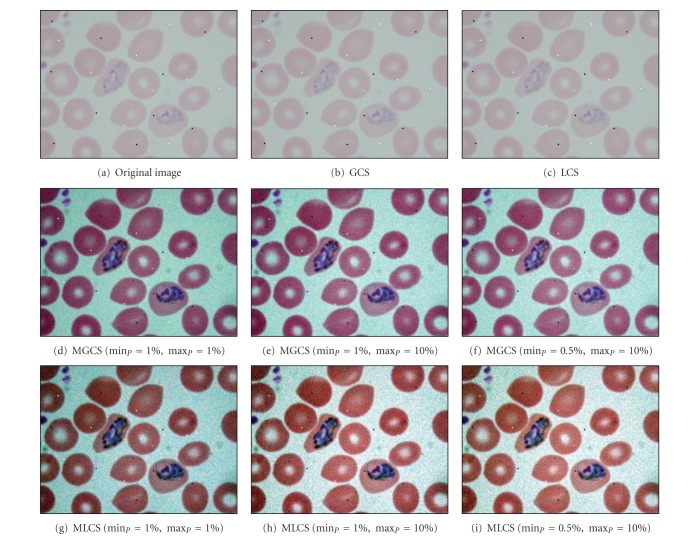
Results of contrast enhancement techniques for the blurred trophozoite image with 0.1% density of salt-and-pepper noise.

Figure 6(a) shows the similar original blurred trophozoite image as shown in Figure 4(a). Here, 0.1% density of salt-and-pepper noise has been added to this original image to measure the effect of contrast enhancement technique on noisy image. The results of applying the four contrast enhancement techniques on malaria images are shown in Figures 6(b)–6(i). Based on Figure 6, image (b) shows the result after applying the GCS technique. Based on the resultant image, there is no sign of enhancement in the image. As previously mentioned, the contrast stretching process depends directly on the minimum and maximum values of the RGB inside the malaria image. After adding the salt-and-pepper noise to the image, the minimum and maximum values have been changed. Since the minimum and maximum values are 0 and 255, respectively, there is no enhancement occur which leads to producing the similar original image. The similar effect can also be seen after applying the LCS technique as shown in image (c). Thus, both GCS and LCS techniques failed to provide the enhanced image if either the minimum value = 0 or maximum value = 255 has been used during the contrast stretching process.

**Figure 7:**
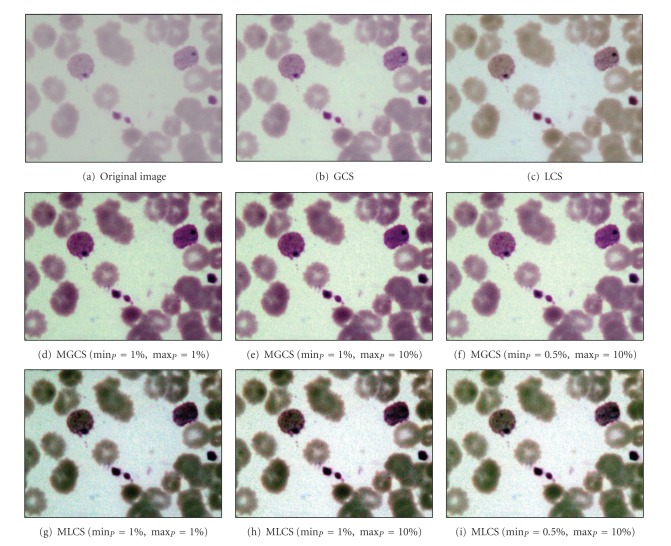
Results of contrast enhancement techniques for the normal ring image.

**Figure 8:**
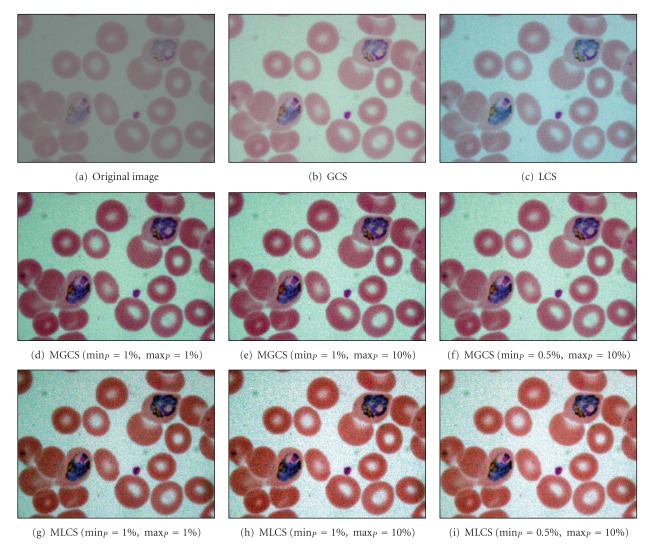
Results of contrast enhancement techniques for the underexposed trophozoite image.

**Figure 9:**
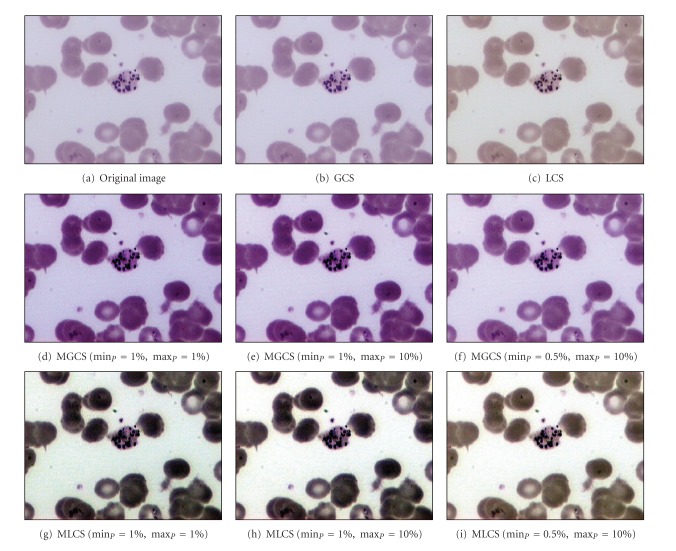
Results of contrast enhancement techniques for the normal schizont image.

**Figure 10:**
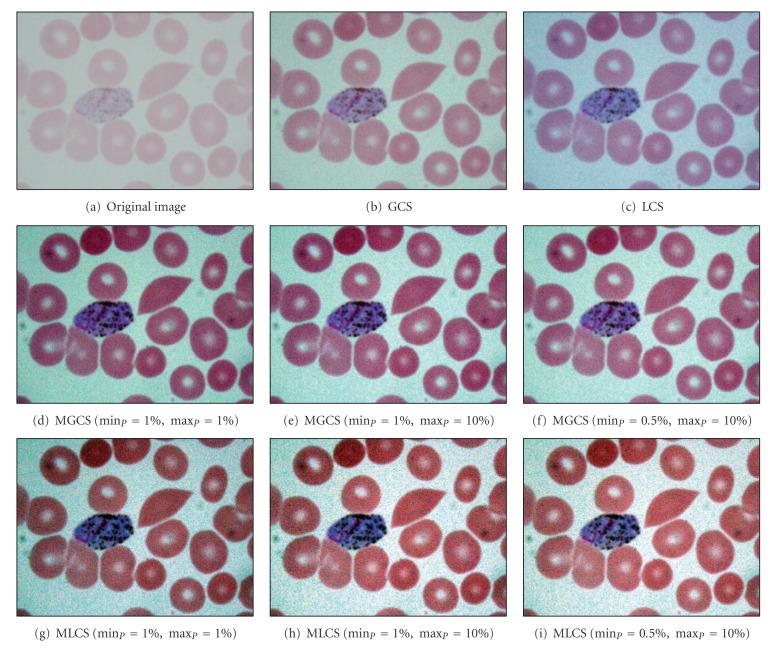
Results of contrast enhancement techniques for the blurred gametocyte image.

 The results obtained after applying the proposed MGCS and MLCS techniques on malaria images are shown in Figures 6(d)–6(f) and Figures 6(g)–6(i), respectively. Based on the resultant malaria images, the images have been simply enhanced by adjusting the minimum and maximum values that lie in a certain percentage of pixels from the total number of pixels in the image. Thus, the contrast of the parasites, infected RBC and background regions has been improved significantly even with the additional of salt-and-pepper noise. The performance of the proposed contrast enhancement techniques has also been tested on other malaria images with three different conditions. The images are called Normal Ring, underexposed trophozoite, normal schizont, blurred gametocyte and Underexposed Gametocyte as shown in Figures 7, 8, 9 and 10, respectively.

Based on the resultant images provided by the four contrast enhancement techniques, GCS technique has not produced satisfactory results due to the slight increase of malaria image contrast. This is mainly because pixels with extreme differences in RGB levels tend to be irregularly scatted in the images, while pixels with the similar RGB levels tend to bunch. Thus, the dynamic adjustment range of contrast enhancement is very limited for the GCS technique but not for the LCS technique. Based on the resultant images, both MGCS and MLCS techniques have produced colour images with good image contrast compared to the conventional GCS and LCS techniques. The previous darker background region has become brighter. Thus, the size and form of the parasites, the size of the RBC, as well as the presence of malarial pigment inside the parasite can be easily seen.

 By comparing both techniques in terms of contrast performance, it has been found that the contrast of the image provided by MLCS technique is slightly better compared to the MGCS technique, since the human vision is mainly sensitive to the local contrast. The application of MLCS technique will result in obtaining different colour appearance of the image because every pixel in the image has been mapped according to the local features of the image. Based on the colour differences, the appearance of the parasite can easily be distinguished from the RBC and background regions. However, this technique has caused a lot of changes in colour for certain malaria images. Based on the resultant images in Figure 7, the colour of the parasite has been changed to dark purple, while the colour of the RBC region has been changed to green after applying the MLCS technique. As a result, the physical appearances of the parasite, RBC and background regions are totally different from the original image. As for the MGCS technique, the overall appearance of images remains mostly unchanged and the enhancement is achieved within the available dynamic range. The images are simply enhanced by adjusting the minimum and maximum values that lie in a certain percentage of pixels, even though every pixel in the image has been mapped in the same way which is independent from the value of surrounding pixels in the image. As a result of applying the MGCS technique, the malaria image becomes clearer and the colour structure does not become disrupted; hence, it can give detailed information about the image.

### 4.2. Quantitative Analysis

 After the four contrast enhancement techniques have been applied on malaria images, the performance of the proposed contrast enhancement techniques is further evaluated by using the distribution separation measure.  Table 1 represents the DSM for GCS and MGCS techniques that have been applied on 6 malaria images, while Table 2 represents the DSM for LCS and MLCS techniques that have been applied on 6 malaria images. 

Based on the average RGB results in Table 1, the DSM provided by the MGCS technique using the three different sets is higher compared to the GCS technique, with the value of DSM for all images more than 40 except for the Normal schizont image. The results also show that the MGCS technique using Set II has produced the highest DSM value, followed by the results provided by Set III and Set I. Thus, the MGCS technique based on Set II has been chosen to be the best enhancement technique compared to the GCS and MGCS based on Set I and Set III.

 Based on the average RGB results in Table 2, the DSM provided by the MLCS technique is higher compared to the LCS technique, with the value of DSM for all images are more than 40 except for the normal schizont image. The results also show that the MLCS technique using the Set I has produced the highest DSM value, followed by the results provided by Set II and Set III. Thus, the MLCS technique based on Set I has been chosen to be the best enhancement technique compared to the LCS and MLCS based on Set II and Set III. Based on the results in Tables 1 and 2, both MGCS and MLCS techniques have produced the more promising results compared to the conventional GCS and LCS techniques.

**Figure 11:**
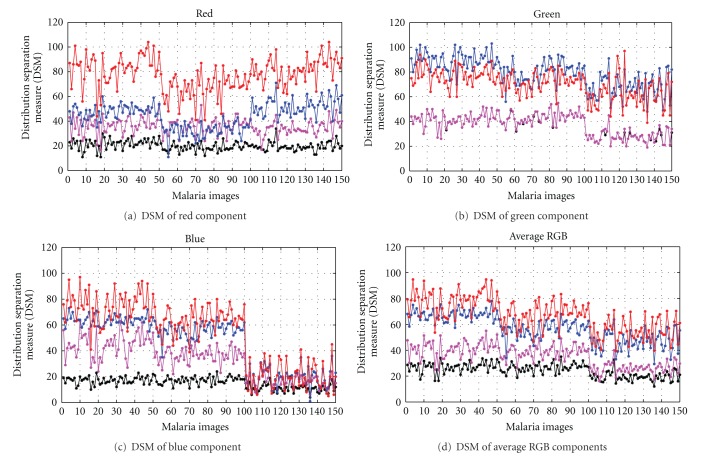
The DSM for the four contrast enhancement techniques that have been applied on 150 malaria images.

 The analysis of DSM for each contrast enhancement technique has also been conducted using the 150 malaria images that have been captured with normal, blurred, and underexposure conditions from different malaria blood slides. Here, MGCS based on Set II and MLCS based on Set I have been chosen for this analysis because each enhancement technique has produced the highest DSM value as shown in Tables 1 and 2, respectively.  Figure 11 graphically illustrates the DSM for the four contrast enhancement techniques that have been applied on 150 malaria images. Based on the axis of malaria images, images 1 till 50 are the ranges for normal image, images 51 till 100 are the ranges for underexposed image, while images 101 till 150 are the ranges for blurred image. The DSMs for the four contrast enhancement based on normal, underexposed, blurred and overall malaria images have also been tabulated in Table 3.

 By referring to Figures 11(a)–11(c), there are separations of DSM values that can be seen between the four contrast enhancement techniques. Based on the results in Table 3, the MLCS technique has provided the highest DSM value for the red and blue components analyses compared to the other contrast enhancement techniques. Meanwhile, the MGCS technique has provided the highest DSM value for the green component analysis. The results of DSM for the average RGB components are graphically illustrated in Figure 11(d). Based on this analysis, there are separations of DSM values that can be seen between the four contrast enhancement techniques. By calculating the average of DSM using the overall 150 images, MLCS technique has proven to be the best with DSM for the average RGB components of 67.17. This is followed by the MGCS, LCS, and GCS techniques with DSM for the average RGB components of 56.27, 35.38, and 24.20, respectively. Based on the high DSM value provided by the MLCS technique, this result has strongly supported the qualitative findings provided in Section 4.1. The DSMs provided by the three different sets of images have also been compared in order to determine the type of image that can give the best enhancement performance. By comparing the DSM for the average RGB components as shown in Table 3, normal image has produced the highest DSM value, followed by the results provided by the underexposed and blurred images. Overall, based on the high values of DSM that have been obtained, both MGCS and MLCS techniques have provided good contrast performance compared to the conventional GCS and LCS techniques. These results are strongly supported by the qualitative findings provided in Section 4.1.

## 5. Conclusions

In this paper, the results of applying the four contrast enhancement techniques namely global, linear, modified global, and modified linear contrast stretching have been presented. Through the experiments using 150 malaria images, the results produced by these four techniques are acceptable in terms of visual quality. The difference between global and linear contrast stretching is the intensity distribution of the image being enhanced. Overall, the proposed MGCS and MLCS techniques have been shown to be good for enhancing the contrast and brightness of the image compared to the conventional GCS and LCS techniques. This statement is strongly supported based on both qualitative and quantitative findings. Here, the MLCS technique has proven to be the best with DSM for the average RGB components of 67.17. This is followed by the MGCS, LCS, and GCS techniques with DSM for the average RGB components of 56.27, 35.38 and 24.20. Based on the qualitative findings provided in Section 4.1, the contrast of the malaria images that have been captured with normal, blurred, and underexposure conditions from different malaria blood slides has been successfully enhanced. As for the noisy image, it has been shown that both GCS and LCS techniques failed to provide the enhanced image if either the minimum value = 0 or maximum value = 255 has been used during the contrast stretching process. However, the proposed MGCS and MLCS techniques manage to overcome this problem by adjusting the minimum and maximum values that lie in a certain percentage of pixels in the image. By comparing the DSM for the proposed contrast enhancement techniques, it has been proven that the contrast of the image provided by MLCS technique is better compared to the MGCS technique. However, the MGCS technique has also shown to be good in enhancing the contrast of the image by keeping the colour structure of the original image, hence it can preserve as much information as the original. Thus, the results significantly demonstrate the suitability of the proposed contrast enhancement techniques in increasing the contrast of the parasites and the infected RBC so that the resultant images would become useful to microbiologists for further analysis of the various stages and species of malaria.

## Figures and Tables

**Table 1:** The DSM for GCS and MGCS techniques that have been applied on 6 malaria images.

Images	Contrast enhancement techniques	Distribution separation measure (DSM)			
		Red	Green	Blue	Average
Blurred trophozoite	GCS	21	31	15	22.33
	MGCS Set I	47	68	29	48.00
	MGCS Set II	54	77	33	**54.67**
	MGCS Set III	51	73	32	52.00

Blurred trophozoite with additional salt-and-pepper noise	GCS	5	5	5	5.00
	MGCS Set I	45	66	29	46.67
	MGCS Set II	53	75	33	**53.67**
	MGCS Set III	49	70	31	50.00

Normal ring	GCS	18	32	18	22.67
	MGCS Set I	39	77	39	51.67
	MGCS Set II	45	89	46	**60.00**
	MGCS Set III	41	81	42	54.67

Underexposed trophozoite	GCS	27	40	22	29.67
	MGCS Set I	58	82	45	61.67
	MGCS Set II	67	94	51	**70.67**
	MGCS Set III	61	89	47	65.67

Normal schizont	GCS	6	5	5	5.33
	MGCS Set I	27	25	32	**28.00**
	MGCS Set II	30	28	24	27.33
	MGCS Set III	28	28	28	**28.00**

Blurred gametocyte	GCS	28	37	17	27.33
	MGCS Set I	55	72	31	52.67
	MGCS Set II	63	82	35	**60.00**
	MGCS Set III	58	78	34	56.67

**Table 2:** The DSM for LCS and MLCS techniques that have been applied on 6 malaria images.

Images	Contrast enhancement techniques	Distribution separation measure (DSM)			
		Red	Green	Blue	Average
Blurred trophozoite	LCS	37	31	23	30.33
	MLCS Set I	75	65	39	**59.67**
	MLCS Set II	66	52	20	46.00
	MLCS Set III	60	49	19	42.67

Blurred trophozoite with additional salt-and-pepper noise	LCS	5	5	5	5.00
	MLCS Set I	71	64	39	**58.00**
	MLCS Set II	64	51	19	44.67
	MLCS Set III	59	46	18	41.00

Normal ring	LCS	23	32	40	31.67
	MLCS Set I	67	79	80	**75.33**
	MLCS Set II	52	64	64	60.00
	MLCS Set III	46	53	57	52.00

Underexposed trophozoite	LCS	41	40	28	36.33
	MLCS Set I	89	80	58	**75.67**
	MLCS Set II	85	73	48	68.67
	MLCS Set III	76	67	44	62.33

Normal schizont	LCS	9	8	13	10.00
	MLCS Set I	35	36	33	**34.67**
	MLCS Set II	13	12	10	11.67
	MLCS Set III	11	9	10	10.00

Blurred gametocyte	LCS	41	41	19	33.67
	MLCS Set I	88	70	37	**65.00**
	MLCS Set II	84	59	20	54.33
	MLCS Set III	75	55	19	49.67

**Table 3:** The DSM for the four contrast enhancement based on normal, underexposed, blurred, and overall malaria images.

Images	Contrast enhancement techniques	Distribution separation measure (DSM)			
		Red	Green	Blue	Average
50 normal images	GCS	21.84	41.70	17.26	26.93
	LCS	36.92	41.72	45.32	41.32
	MGCS Set II	48.22	**89.90**	63.62	67.25
	MLCS Set I	**83.35**	77.40	**74.96**	**78.57**

50 underexposed images	GCS	18.84	43.20	17.32	26.45
	LCS	36.38	43.32	36.46	38.72
	MGCS Set II	32.90	**80.08**	55.92	56.30
	MLCS Set I	**67.59**	72.22	**64.06**	**67.96**

50 blurred images	GCS	19.50	27.34	10.78	19.21
	LCS	34.34	27.80	16.18	26.11
	MGCS Set II	50.36	**66.26**	19.18	45.27
	MLCS Set I	**82.19**	62.10	**20.68**	**54.99**

Overall 150 images	GCS	20.06	37.41	15.12	24.20
	LCS	35.88	37.61	32.65	35.38
	MGCS Set II	43.83	**78.75**	46.24	56.27
	MLCS Set I	**77.71**	70.57	**53.23**	**67.17**

## References

[1] WHO (2010). *Basic Malaria MicroScopy, Part I. Learner’s Guide*.

[2] Beare NAV, Taylor TE, Harding SP, Lewallen S, Molyneux ME (2006). Malarial retinopathy: a newly established diagnostic sign in severe malaria. *American Journal of Tropical Medicine and Hygiene*.

[3] White NJ (2008). Plasmodium knowlesi: the fifth human malaria parasite. *Clinical Infectious Diseases*.

[4] (2010). *World Malaria Report 2010*.

[5] Makler MT, Palmer CJ, Ager AL (1998). A review of practical techniques for the diagnosis of malaria. *Annals of Tropical Medicine and Parasitology*.

[6] Zou LH, Chen J, Zhang J, García N Malaria cell counting diagnosis within large field of view.

[7] Ross NE, Pritchard CJ, Rubin DM, Duse AG (2006). Automated image processing method for the diagnosis and classification of malaria on thin blood smears. *Medical & Biological Engineering & Computing*.

[8] Mitiku K, Mengistu G, Gelaw B (2003). The reliability of blood film examination for malaria at the peripheral health unit. *The Ethiopian Journal of Health Development*.

[9] Chatterji BN, Murthy RN Adaptive contrast enhancement for colour images.

[10] Preethi SJ, Rajeswari K (2010). Image enhancement techniques for improving the quality of colour and gray scale medical images. *International Journal on Computer Science and Engineering*.

[11] Aimi Salihah AN, Mashor MY, Harun NH, Rosline H Colour image enhancement techniques for acute leukaemia blood cell morphological features.

[12] Saleh MD, Eswaran C, Mueen A (2010). An automated blood vessel segmentation algorithm using histogram equalization and automatic threshold selection. *Journal of Digital Imaging*.

[13] Tong HL, Fauzi MFA, Haw SC Automated hemorrhage slices detection for CT brain images.

[14] Tek FB (2007). *Computerised diagnosis of malaria [Ph.D. thesis]*.

[15] Hanif NSMM, Mashor MY, Mohamed Z Image enhancement and segmentation using dark stretching technique for Plasmodium Falciparum for thick blood smear.

[16] DPDx Comparison of plasmodium species which cause malaria in humans. http://www.dpd.cdc.gov/dpdx/html/malaria.htm.

[17] Arici T, Altunbasak Y Image local contrast enhancement using adaptive non-linear filters.

[18] Matkovic K, Neumann L, Neumann A, Psik T, Purgathofer W Global contrast factor—a new approach to image contrast.

[19] Sahidan SI, Mashor MY, Wahab ASW, Salleh Z, Jaafar H Local and global contrast stretching for color contrast enhancement on ziehl-neelsen tissue section slide images.

[20] Ngah UK, Ooi TH, Khalid NEA, Venkatachalam PA The seed based region growing image processing with embedded enhancement techniques.

[21] Singh S, Al-Mansoori R (2000). Identification of regions of interest in digital mammograms. *Journal of Intelligent Systems*.

[22] Singh S, Bovis K (2005). An evaluation of contrast enhancement techniques for mammographic breast masses. *IEEE Transactions on Information Technology in Biomedicine*.

